# Comparison of two teaching methods for stopping the bleed: a randomized controlled trial

**DOI:** 10.1186/s12909-022-03360-4

**Published:** 2022-04-14

**Authors:** Shuangyi Chen, Jinfei Li, Michael A. DiNenna, Chen Gao, Shijie Chen, Song Wu, Xiaohong Tang, Jinshen He

**Affiliations:** 1 Department of Orthopaedic Surgery, The Third Xiangya Hospital of Central South University, Changsha, 410013 China; 2 Xiangya School of Medicine, Central South University, Changsha, 410013 China; 3 Department of Mechanical and Material Science Engineering, University of Pittsburgh, Pittsburgh, PA 15213 USA; 4 Department of Transplantation, The Second Xiangya Hospital of Central South University, Changsha, 410013 China; 5 Clinical Skills Training Center, The Third Xiangya Hospital of Central South University, Changsha, 410013 China

**Keywords:** PTEBL, Caesar (trauma patient simulator), Outstanding doctoral candidates, Stop the Bleed, Traumatic hemostasis, Educational reform

## Abstract

**Background:**

The “Stop the Bleed” (STB) campaign has achieved remarkable results since it was launched in 2016, but there is no report on the teaching of an STB course combined with a trauma patient simulator. This study proposes the “problem-, team-, and evidence-based learning” (PTEBL) teaching method combined with Caesar (a trauma patient simulator) based on the STB course and compares its effect to that of the traditional teaching method among outstanding doctoral candidates training in haemostasis skills.

**Method:**

Seventy-eight outstanding doctoral candidate program students in five and eight-year programs were selected as the research subjects and were randomly divided into a control group (traditional teaching method, *n* = 34) and an experimental group (PTEBL teaching method combined with Caesar, *n* = 44). Their confidence in their haemostasis skills and willingness to rescue injured victims were investigated before and after the course in both groups.

**Result:**

Students’ self-confidence in their STB skills and the willingness to rescue improved after the class in both groups. Compared with the control group, students in the experimental group were more confident in compressing with bandages and compressing with a tourniquet after a class (compressing with bandages: control group 3.9 ± 0.8 vs. experimental group 4.3 ± 0.7, *P* = 0.014; compressing with a tourniquet: control group 3.9 ± 0.4 vs. experimental group 4.5 ± 0.8, *P* = 0.001) More students in the experimental group than the control group thought that the use of Caesar for scenario simulation could improve learning (control group 55.9% vs. experimental group 81.8%, *P* = 0.024), and using this mannequin led to higher teacher-student interaction (control group 85.3% vs. experimental group 97.7%, *P* = 0.042). The overall effectiveness of the teaching was better in the experimental group than in the control group (control group 85.3% vs. experimental group 97.7%, *P* = 0.042). There was a significant positive correlation between teacher-student interactions and the overall effectiveness of teaching (*R* = 1.000; 95% CI, 1.000–1.000; *P* < 0.001).

**Conclusion:**

The PTEBL teaching method combined with Caesar can effectively improve student mastery of STB skills and overcome the shortcomings of traditional teaching methods, which has some promotional value in the training of outstanding doctoral candidates in STB skills.

**Supplementary Information:**

The online version contains supplementary material available at 10.1186/s12909-022-03360-4.

## Background

Unintentional injury is the leading cause of death among people aged 1–45 years old, resulting in more than 160,000 deaths each year in the United States and showing a gradually increasing trend [1]. Studies show that nearly 60% of potentially survivable deaths are caused by haemorrhaging, which means that controlling bleeding in a timely and effective manner is the key to preventing death in injury patients [2, 3]. The US military was the first to make a breakthrough in the study of traumatic haemostasis and has reduced battlefield mortality by 44.2% over the 16 years of war in Iraq and Afghanistan due to its medical advancements in the field of prehospital haemorrhage control [4]. A National Academies of Sciences, Engineering, and Medicine Report recommended that civilians deserved the care and improvement benefits achieved through military medicine [5]. Thus, the White House launched a national public awareness campaign, “Stop the Bleed” (STB), in October 2015 to educate and empower the public in bleeding control [6–9]. This campaign has been proven to achieve remarkable results [10]. Since its inception, the movement has gained more than 15,000 instructors in the United States and trained more than 120,000 people across the country [11].

In China, injury is the leading cause of death and disability among the younger population, and the incidence of road traffic-related deaths is significantly higher than average in high- and middle-income countries [12]. However, there is no report on the teaching of STB courses in China, and only some schools use Caesar, a mechanical trauma patient who can simulate a trauma patient and provide real-time feedback of vital signs such as heart rate, blood pressure, and respiration, to improve students’ ability to address emergency and critical diseases [13]. At present, most traditional haemostatic trauma training in China is one-way skill training, which is performed using the three steps "demonstration-exercise-examination". The traditional teaching method contributes to improved operational proficiency. Nevertheless, it cannot effectively improve students' initial diagnosis, decision-making, and correct handling of bleeding due to the complexity of clinical scenes [14]. Therefore, it is essential to introduce STB skills and proper teaching methods that are suitable for medical education in China.

Our team put forward a new teaching method named “problem-, team- and evidence-based learning” (PTEBL) in 2012 [15]. This teaching method emphasizes problem-orientation teamwork and evidence-based decisions to maximize student engagement and encourage interactive learning [16, 17]. Furthermore, in 2018, the Ministry of Education of China put forward “The Opinions on Strengthening the Collaboration between Medicine and Education to Implement the Outstanding Doctoral Candidates Training Program 2.0”, which emphasized a teaching reform of practical skills in the training of outstanding doctoral candidates who were selected from normal medical training programs [18]. Therefore, based on the advantages of the STB course and combined with the teaching experience of our school, we adopted the PTEBL teaching method combined with using Caesar to train outstanding doctoral candidates. We proved the feasibility of this model, which can effectively overcome the shortcomings of the traditional teaching model.

## Methods

### Study design

**Fig. 1:**
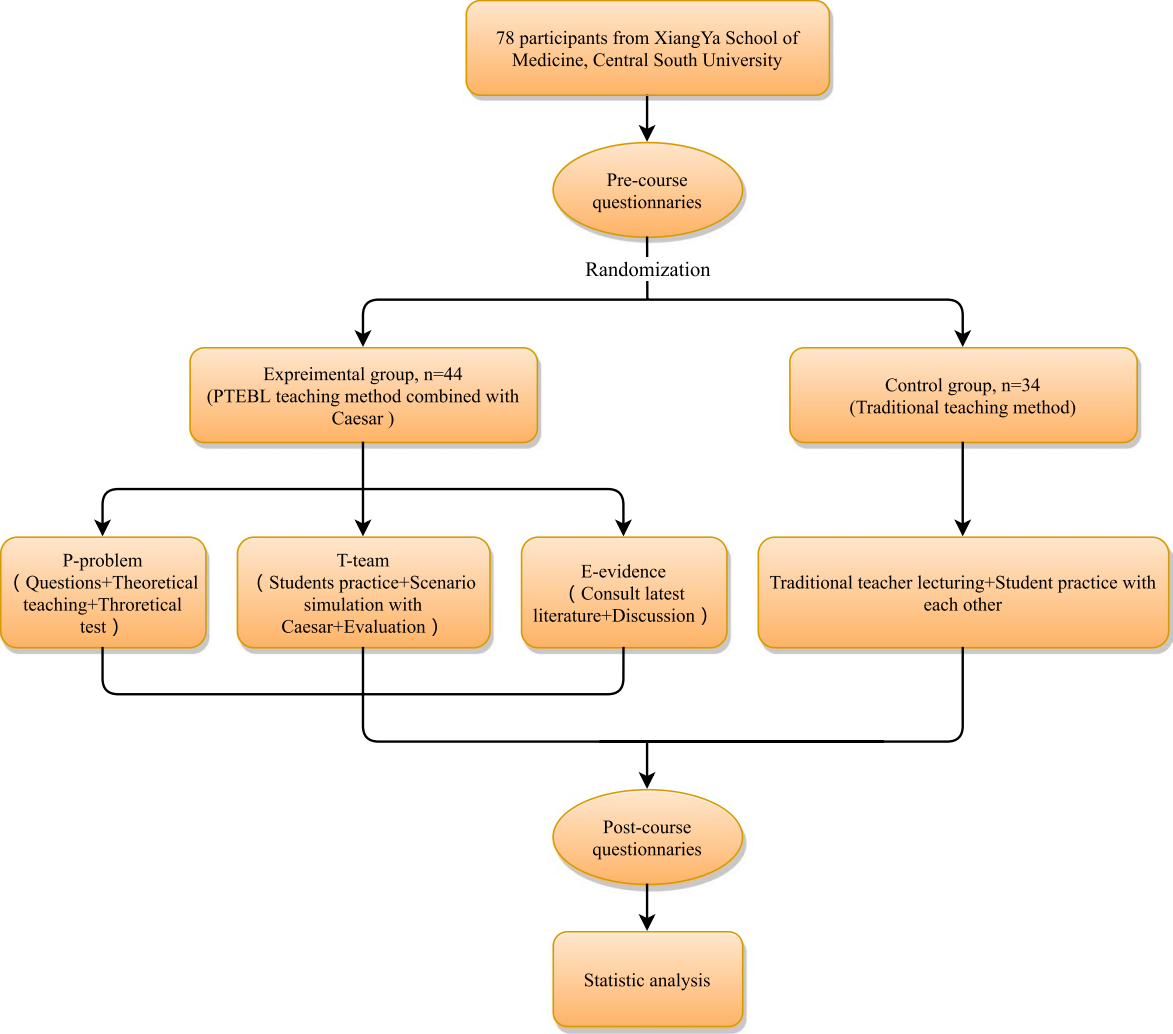
Enrollment, randomization, and protocol of participants

This study is a randomized controlled trial, and the hospital ethics committee approved the research (ID: 2021-S078). All the participants were randomly divided into the experimental group and the control group (Fig. 1). The experimental group was taught using the PTEBL teaching method combined with the use of Caesar, and the control group was taught using the traditional teaching method. Based on the Likert scale and in reference to prior studies [19], a questionnaire (see Additional file 1 and Additional file 2) was administered before and after class. The students were asked about their willingness to rescue a patient at the first scene of traumatic bleeding and their confidence in the three haemostatic skills. The theoretical and operational scores of the experimental group were tested. The above results were used as the criteria for evaluating the effectiveness of teaching the PTEBL teaching method combined with the use of Caesar.

### Study participants and set up

Thirty-nine students from a five-year outstanding doctoral candidate training program were randomly assigned to The Second Xiangya Hospital and The Third Xiangya Hospital at a ratio of 1:1. Since the eight year students in clinical medicine needed to be assigned doctoral supervisors for their future studies and the number of doctoral supervisors available for allocation between The Third Xiangya Hospital and The Second Xiangya Hospital was 1.8:1, 39 eight year students in clinical medicine were randomly assigned to the two hospitals at a ratio of 1.8:1. Among them, there were 34 students at The Second Xiangya Hospital (20 five year students in the outstanding doctoral candidates training program, 14 eight year students in clinical medicine) and 44 students in The Third Xiangya Hospital (19 five year students in the outstanding doctoral candidates training program, 25 eight year students in clinical medicine). Both five-year and eight-year classes belong to the outstanding doctoral candidates training program. Before the experiment, the only difference between the two classes was that the eight-year clinical students had learned more science and engineering foundations than the five-year clinical students, but the medical education progress was the same. An a priori power analysis was performed using G*power v3.1 and showed that the sample size was sufficient to discover potential intergroup and intragroup differences. All participants gave written informed consent. The study grouping and implementation were as follows:

The experimental group (PTEBL teaching method + students practice with Caesar): Forty-four students were in the excellence training plan at The Third Xiangya Hospital. For the experimental group students, we first introduced the background of trauma haemostasis, the core of the PTEBL teaching method, and the use of Caesar, the trauma patient simulator, which was recognized and operated by the students.

The control group (traditional teacher lecture + student practice with each other) included 34 students with excellent training plans at The Second Xiangya Hospital. The conventional model was used for the students in the control group, and the experimental teaching reform was not implemented. A questionnaire was given to the students to evaluate the effect of the traditional teaching.

### Study protocol

For the control group, the teacher explained three STB haemostasis techniques (compress with fingers, bandages, or a tourniquet), highlighting the essentials of haemostasis techniques and giving step-by-step demonstrations. Students then practised these skills with each other and were corrected by the teacher one by one.

For the experimental group, the PTEBL teaching method combined with the use of Caesar was adopted. The implementation plan for this method was as follows:

1. P-problem: In this part, teachers distributed courseware on trauma haemostasis and the relevant authoritative guidelines to students before class. At the beginning of the class, the students were shown a traumatic bleeding scenario in The Good Doctor, which showed a child who was hit by a collapsed billboard and who had ruptured jugular vein bleeding; the doctors on the scene performed emergency haemostasis. The following questions were put forward: (Q1) How do you stop the bleeding effectively? (Q2) Can we perform cardiopulmonary resuscitation directly? (Q3) Which actions were handled well in the video and which were not done well? The students were asked to study under the guidance of the above questions. After the lecture, the teacher distributed classroom tests (see Additional file 3) to the students to test their mastery of the theoretical knowledge of trauma haemostasis. The content of the tests was the steps in trauma haemostasis processing and basic knowledge of STB skills, with a passing score of no less than 60 (full score was 100).
2. T-team: In this part, students were randomly divided into several teams of 2–3 members, who roleplayed the following roles: two doctors (roles A and B) and one family member (role D, which is not available when the team has only two students). After the teacher plays a video introducing Caesar and students have practised hands-on bleeding control skills, a scenario simulation is then performed based on the wounds on Caesar: You and your companions (roles A and B) witnessed a severe car accident. The injured person (role C, Caesar) was conscious, but his popliteal arteries kept spurting blood. What would you do to rescue Caesar? Caesar can provide feedback to inform the students of changes in vital signs and to evaluate the haemostatic effect accurately. Two scenarios were simulated for each group, and each operation was timed. Student fluency and completion of the haemostasis operation were scored to test their mastery of STB operation skills.
3. E-evidence: In this part, the students were asked to diverge in their thinking using materials distributed by teachers, to consult the latest literature, and jointly discuss the latest techniques or concepts of "trauma haemostasis" in class.

The durations of the control and experimental group studies were standardized, and both were 4 h. A questionnaire was conducted before and after the course (see Additional file 1 and Additional file 2), and it addressed student confidence in STB skills, willingness to rescue at the trauma scene and evaluation of the course, etc., to evaluate the effect of teaching in the control group and the experimental group. The competencies needed for medical students in terms of specific standards were established on the basis of the latest International Medical Association guidelines and other related studies [19–25].

### Statistical analysis

Statistical analysis and mapping were performed with SPSS 26.0 (IBM Corp., Armonk, NY, USA) and Prism 9.0 (GraphPad Software, San Diego, CA, USA). A t test was used to analyse the measurement data (mean ± standard deviation), a chi-square test was used to analyse the nominal data, and a rank-sum test was used to analyse the ordinal data. A *P* < 0.05 was regarded as statistically significant. Spearman's correlation coefficient between the independent variables and the results are presented as a correlation heat map. A logistic regression analysis was performed by asking about post-course confidence on three STB skills and post-course willingness as the independent variables and teaching reform, major, teacher enthusiasm, and interaction between teacher and students as the dependent variables. Bonferroni correction was used for multiple testing results.

## Results

### Demographics

A total of 78 participants completed the study, with 34 in the control group and 44 in the experimental group. Here, 78 pre-course and after-course questionnaires were distributed to the subjects, with a return rate of 100%. Through an analysis of the pre-course questionnaire, we found that there was no significant difference in sex, age, haemostatic experience, haemostatic confidence or willingness to rescue between the two groups (*P* > 0.05) (Table 1). Among all the subjects, there were 40 males and 38 females, with an average age of 20 ± 1 years.

**Table 1:** Study participants and demographics

Variable	Experimental group(*n* = 44)	Control group(*n* = 34)	*P* Value
Male, n (%)	21(47.7)	19(55.9)	0.475
Age (years), mean ± SD	20 ± 1	20 ± 1	0.221
No prior training in trauma hemostasis, n (%)	43(97.7)	34(100)	0.808
Have the willingness to stop bleeding in a real-life emergency, n (%)	23(52.3)	16(47.1)	0.648
Number of students who have used compression hemostasis, n(%)	24(54.5)	20(58.8)	0.706
Number of students who have used bandaging hemostasis, n(%)	8(18.2)	5(14.7)	0.683
Number of students who have used tourniquet hemostasis, n(%)	1(2.3)	0	1
Very confident or confident in compressing with fingers, n (%)	14(31.8)	10(29.4)	0.361
Very confident or confident in compressing with bandages, n (%)	7(15.9)	6(17.6)	0.912
Very confident or confident in compressing with a tourniquet, n (%)	8(18.2)	5(14.7)	0.489

*SD* Indicates standard deviation

### Willingness to rescue at the first scene of traumatic bleeding

There was a significant increase in the number of subjects who chose to perform haemostasis at the first scene of traumatic blood loss after training (*P* < 0.001). However, there was no significant difference between the two groups (*P* = 0.660) (Table 2). The difference in the willingness to rescue between the five-year outstanding doctoral candidates’ training program students and the eight-year medical students was statistically significant (*P* = 0.048).

**Table 2:** Willingness to rescue at the first scene of trauma bleeding

Variable	N (%) of Participants(*n* = 78)						*P* Value^+^
	Experimental group, n (%)			Control group, n (%)			
	Pre-course	Post-course	*P* Value^a^	Pre-course	Post-course	*P* Value^a^	
Have the willingness to stop bleeding in a real-life emergency	23 (52.3)	39 (88.6)	< 0.001	16 (47.1)	32 (94.1)	< 0.001	0.660

^a^Comparison between the pre-course and the post-course

^+^Comparison between the experimental group and the control group post-course

### Confidence in various haemostatic skills

The students’ self-confidence in compressing with fingers, bandages, or a tourniquet after class in both groups was statistically higher than that before the class (*P* < 0.001). In compressing with bandages and compressing with a tourniquet, the average self-confidence of the students in the control group was 3.9 ± 0.8 and 3.9 ± 0.4, respectively, while that in the experimental group was 4.3 ± 0.7 and 4.5 ± 0.8, respectively, which was significantly higher than that in the control group (*P* = 0.014 and 0.001, respectively). These data are presented in Table 3. There was no significant difference in the students’ confidence in performing the three haemostatic methods between the five-year outstanding doctoral candidate training program students and the eight-year clinical medicine students (*P* = 0.567, 0.877 and 0.915, respectively).

**Table 3:** Confidence in various hemostatic methods

Variable (Very comfortable or comfortable)	n(%) of Participants(*n* = 78)						*P* Value^+^
	Experimental group, n(%)			Control group, n(%)			
	Pre-course	Post-course	*P* Value^a^	Pre-course	Post-course	*P* Value^a^	
Compressing with fingers	14 (31.8)	30 (68.2)	< 0.001	10 (29.4)	23 (67.6)	< 0.001	0.545
Compressing with bandages	7 (15.9)	38 (86.4)	< 0.001	6 (17.6)	22 (64.7)	< 0.001	0.014
Compressing with a tourniquet	8 (18.2)	37 (84.1)	< 0.001	5 (14.7)	22 (64.7)	< 0.001	0.001

^a^Comparison between the pre-course and the post-course

+ Comparison between the experimental group and the control group post-course

### Scores and scenario simulation results for the experimental group

All 44 students who participated in the class theory test of this course passed the test (scores no less than 60 were passing) with a pass rate of 100%, and the average score was 97.1 ± 5.28. In the final stage of the haemostasis operation with Caesar, all groups achieved the treatment task, which made the vital signs of the trauma patient stable. The scores and time consumption for the two scenario simulations in each group are shown in Table 4. The score for the second scenario simulation was significantly improved compared with the first one (*P* < 0.001). Similarly, the time consumption in the second scenario simulation was significantly less than that of the first one (*P* = 0.001).

**Table 4:** scenario simulation results of experimental group

Variable	The first scenario simulation	The second scenario simulation	*P* Value
Average score (points)	91.7 ± 2.6	95.7 ± 2.6	< 0.001
Average time (seconds)	255.4 ± 49.4	204.3 ± 37.2	0.001

### Students’ approval of improvements in various abilities

**Fig. 2:**
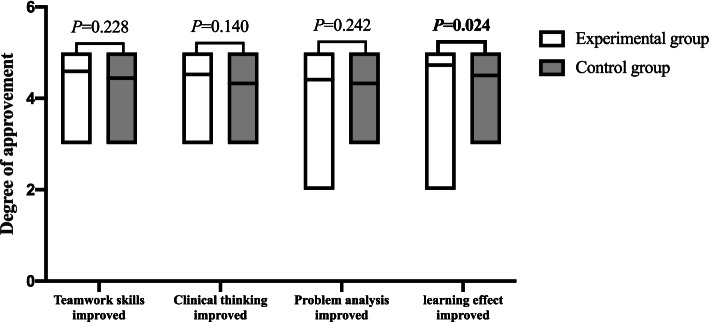
Students' approval of improvements in various abilities

After attending the course, 81.8% (36/44) of students in the experimental group noted that the scenario simulation improved their traumatic haemostasis learning, while in the control group, only 55.9% (19/34) of students agreed that the operation improved their traumatic haemostasis learning, and the difference was statistically significant (*P* = 0.024). In terms of teamwork skills, clinical thinking and problem analysis, more than 80% of the students in both groups agreed that their abilities had improved, and there was no significant difference between the two groups (*P* = 0.228, 0.140, and 0.242, respectively). The degrees of student improvement in various abilities are shown in Fig. 2.

### Evaluation of teaching effectiveness

**Fig. 3:**
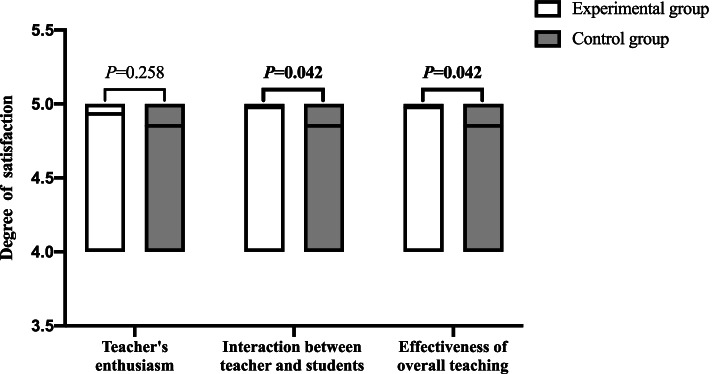
Students' satisfaction with teaching effectiveness

All the participants were satisfied or very satisfied with the teachers' enthusiasm, the interaction between teachers and students, and the overall effectiveness of the teaching. A total of 93.2% (41/44) and 85.3% (29/34) of the students in the experimental group and the control group, respectively, were very satisfied with the teacher's enthusiasm for teaching, and there was no significant difference between the two groups (*P* = 0.258). A total of 97.7% (43/44) and 85.3% (29/34) of the students in the experimental group and the control group, respectively, were very satisfied with the interaction between teachers and students, and the difference was statistically significant (*P* = 0.042). After the course training, 97.7% (43/44) of the students in the experimental group were very satisfied with the overall effectiveness of the teaching, while only 85.3% (29/34) of the students in the control group were very satisfied with the overall effectiveness of the teaching, which was statistically lower than that in the experimental group (*P* = 0.042). The proportions of students who were very satisfied with various variables are shown in Fig. 3.

### Correlation heatmap of relevant independent variables

**Fig. 4:**
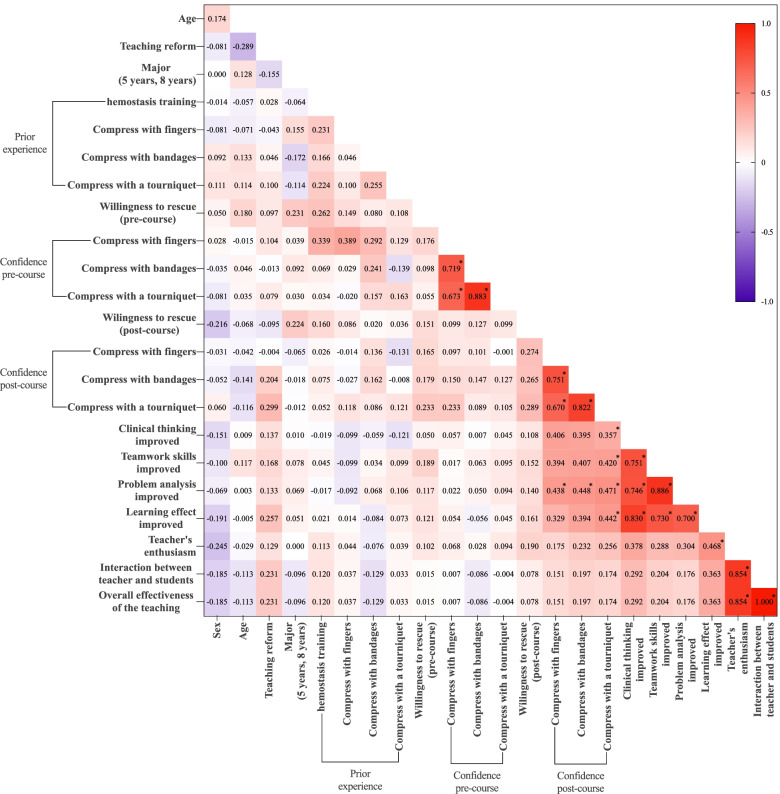
Correlation heatmap of relevant independent variables. *: *P* < 0.0002 level of significance after Bonferroni correction of 253 × test

A Spearman correlation analysis was used to analyse the correlation of independent variables. Through the analysis, we found that there was a significant correlation between the students’ confidence in their knowledge of the three haemostatic methods pre-course, between the students’ confidence in their knowledge of the three haemostatic methods post-course, between their improvement in the four abilities, and between their satisfaction with the three variables of teaching effectiveness (|*r*|> 0.6, *P* < 0.0002). Among the groups, 7 groups of variables were highly correlated (|*r*|> 0.8, *P* < 0.0002). The highest positive correlation was between the overall effectiveness of the teaching and interaction between teachers and students (*R* = 1.000; 95% CI, 1.000–1.000; *P* < 0.0002). Then, the second highest positive correlation was between problem analysis improvement and teamwork skills improvement (*R* = 0.886; 95% CI, 0.753–0.956; *P* < 0.0002). The results are shown in Fig. 4.

### Regression analysis of post-course confidence and willingness to rescue

As shown in Table 5, we found that the regression relationship between teaching reform and confidence in compressing with a tourniquet post-course was statistically significant (β = 1.229, 95% CI, 0.327–2.131, *P* = 0.008), and the use of the PTEBL teaching method combined with Caesar was a positive factor for increasing confidence in compressing with a tourniquet post-course.

**Table 5:** Regression analysis of post-course confidence and willingness to rescue

	Compress with fingers (Confidence post-course)		Compress with bandages (Confidence post-course)		Compress with a tourniquet (Confidence post-course)		Willingness to rescue (post-course)	
	β (95% CI)	*P* value	β (95% CI)	*P* value	β (95% CI)	*P* value	β (95% CI)	*P* value
Teaching reform	-0.113 (-0.975, 0.748)	0.796	0.740 (-0.150, 1.631)	0.103	1.229 (0.327, 2.131)	0.008^*^	0.499 (-2.397, 1.399)	0.606
Major (5 years, 8 years)	0.258 (-0.581, 1.098)	0.547	0.039 (-0.814, 0.893)	0.928	0.073 (-0.787, 0.934)	0.867	1.835 (-4.075, 0.429)	0.112
Teacher's enthusiasm	1.010 (-1.691, 3.712)	0.464	1.709 (-1.005, 4.422)	0.217	2.807 (0.117, 5.497)	0.041	1.811 (-4.828, 1.180)	0.235
Interaction between teacher and students	0.143 (-3.000, 3.258)	0.929	-0.421 (-3.555, 2.713)	0.792	-1.961 (-1.111, 5.032)	0.211	-0.306 (-3.576, 4.183)	0.877

^*^P < 0.0125 level of significance after Bonferroni correction of 4 × test

## Discussion

Injury is the leading cause of death among young adults. The key element in the death of injury patients is failure to control bleeding in a timely and effective manner. For this reason, many projects, such as the STB campaign, the Hartford Consensus, and the Federal Emergency Management Agency’s "You are the Help until Help arrives", are calling for the strengthening of trauma haemostasis education for the general public [6, 8, 9, 26]. The STB campaign has been implemented for six years and has achieved remarkable results. Research by AlSabah et al. shows that nearly 90% of participants said that the STB campaign contributed to promoting health and improving personal safety awareness [27]. Schroll et al. [28] pointed out that the traumatic haemostasis course is important for teaching medical students. Research by Sarah Beth Dinwiddie et al. [29] also showed that STB training is effective at improving students’ knowledge and confidence. However, the traditional teaching method of one-way skill training is still used in China at present. This approach is taught only through teachers' explanations and students' personal operations and cannot be effectively integrated with clinical practice. For the education of outstanding doctoral candidates, more attention should be given to students’ innovation, exploration, and cooperation abilities. Research by Faisal et al. [30] shows that problem-based learning is more helpful in training medical students than traditional lecture-based learning. Burgess et al. [31] introduced team-based learning on the basis of problem learning, and Chakraborti et al. [32] introduced evidence-based learning on the basis of team learning, both of which have been shown to achieve beneficial results in medical education. Based on the above reasons, we hope to introduce STB courses suitable for the training of outstanding doctoral candidates to make up for the shortcomings of traditional teaching methods. However, the traditional STB course also has drawbacks during the implementation process. The research of Villegas et al. [33] shows that people reported overwhelmingly that the model is not authentic enough. If the training is more realistic, it will be more effective. Zwislewski et al. [34] also emphasized the importance of hands-on training in STB skill learning. Therefore, we adopted the PTEBL teaching method combined with Caesar, the trauma patient simulator, for the STB skills training of outstanding doctoral candidates.

In this study, all the students in the experimental group passed the theoretical test and the scenario simulation test. They also significantly improved their confidence in the three basic skills of the STB course and their willingness to rescue at the first scene of traumatic bleeding. This finding shows that this teaching method can effectively teach STB skills and help students master and implement them. In the scenario simulation test, the second scenario simulation takes less time than the first scenario simulation, and the average performance was improved. This observation shows that two training sessions are more effective than single training sessions, which can improve the speed and quality of trauma haemostasis operations, thereby reducing the amount of bleeding by patients and helping to improve the treatment success rate. This result affirms the training effect of teamwork and scenario simulation on students' mastery and proficiency in STB skills. In our research, we found that although using traditional teaching methods could improve students’ confidence in various haemostasis skills, students’ confidence was still low after the class. This feature is more prominent when they were compressing with bandages and compressing with a tourniquet technique, which may be related to the fact that traditional one-way skill training does not allow students to use these two more difficult operations flexibly. The experimental group's confidence in compressing with bandages and compressing with a tourniquet technique after the class was significantly higher than that of the control group. In addition, the regression analysis results also show that the new teaching method has a positive effect on improving students' confidence in compressing with a tourniquet after class, which indicates that the PTEBL teaching method can effectively improve students' mastery of haemostasis skills and can also make up for the shortcomings of traditional teaching methods. This finding is closely related to the superiority of STB courses. The research of Ali et al. has shown that the STB course can promote the correct placement of tourniquets and increase comfort levels in 75% of students [35], which is consistent with the results of our research. In addition, we found that there was a significant difference in willingness to rescue the injured post-course between five-year and eight-year clinical students, indicating that this method may be more helpful at improving the willingness to rescue traumatic bleeding patients at the first scene by students of the eight year program in clinical medicine. However, since the regression analysis did not find a regression relationship between the major (five-year program and eight-year program) and willingness to rescue post-course, this result requires further experimental verification. In addition, in the after-school questionnaire, we found that compared with the traditional teaching method for students’ autonomous operation, students responded that the use of Caesar for scenario simulation could improve the effectiveness of learning, which is consistent with the results of Villegas’s research [33]. We believe that the use of Caesar for more realistic scenario simulations is also one of the reasons for the experimental group's higher confidence in compressing with bandages and compressing with a tourniquet technique. Based on this result, we speculate that the use of Caesar alone can also have a better impact on improving the teaching effect of the traditional teaching method, but this conclusion must be supported by further experiments.

The heat map shows that the degree of interaction between teachers and students is highly correlated with the overall effectiveness of the teaching. The data here are rather unique. We found that all students gave the same rating to the overall teaching effect and the teacher-student interaction, indicating that students’ mastery of skills largely depends on teacher-student interaction. However, since this heat map used a large number of correlations, which may lead to a high false-positive rate, this analysis should only be used for hypothesis generation. Burgess et al. [36] showed that team-based learning could improve student participation in the course. The PTEBL teaching method uses teamwork as the core and increases the communication between students and teachers. As shown in Fig. 3, when the teachers’ enthusiasm does not show a significant difference, the PTEBL teaching method combined with Caesar can effectively improve the interaction between teachers and students, thereby further improving the overall effectiveness of the teaching.

In summary, the PTEBL teaching method combined with Caesar has achieved remarkable results. However, both the traditional method and the PTEBL method can improve student confidence in haemostatic skills and the willingness to rescue. The PTEBL method improves some of the students' trauma haemostatic skills to a greater extent and can create a better teaching atmosphere and achieve better effectiveness in teaching STB skills, which is consistent with our expected results.

However, this study and the course still have some limitations. For example, the random assignment was not strict, and the sample size was not large enough, which may have some impact on the reliability of the results. The results obtained in the form of a questionnaire are not comprehensive enough to allow for a comparative self-assessment with an evaluation of the learning gain. In addition, the teaching mode of using Caesar alone in traditional teaching methods was not performed, so the influence of using Caesar alone on the teaching effect cannot be accurately evaluated. The cost of Caesar, the trauma patient, is relatively high and difficult to obtain, which imposes some restrictions on the implementation of teaching. In addition, many studies have shown that although the STB course can effectively improve students’ trauma haemostatic skills, the retention of these skills was poor [7, 37, 38]. Therefore, increasing the retention of skills is extremely important for improving the mastery rate of trauma haemostasis skills. In addition, research by Dhillon et al. [39] showed that although STB courses can achieve decent results, the general public’s acquisition rate of necessary equipment for these skills is low. The cost, time and accessibility of items during an event are still the most common obstacles. Therefore, to improve the general public’s willingness to treat bleeding patients, we must not only strengthen trauma haemostasis education but also make it so that people can easily obtain the required materials for a traumatic bleeding situation. Therefore, the teaching reform of STB skills can be further explored.

## Conclusion

This study evaluates the effectiveness of using the PTEBL teaching method combined with Caesar, the trauma patient simulator, and the traditional teaching method on the training of outstanding doctoral candidates for the first time. In this study, the PTEBL teaching method combined with Caesar can effectively improve students' mastery of traumatic haemostasis skills and can simultaneously make up for the shortcomings in the traditional teaching method, which has promotional significance in STB skills training for outstanding doctoral candidates.

## Supplementary Information

**Additional file 1.**

**Additional file 2.**

**Additional file 3.**

## Data Availability

The datasets used and/or analyzed during the current study are available from the corresponding author on reasonable request.

## Abbreviations

STB: Stop The Bleed
PTEBL: Problem-, team-, and evidence-based learning
SD: Standard Deviation
