# Supplementary material for: Comparison of two teaching methods for stopping the bleed: a randomized controlled trial
Source: BMC Med Educ. 2022 Apr 14;22:281. doi: 10.1186/s12909-022-03360-4 (PMC9009024; doi:10.1186/s12909-022-03360-4)
Supplement: Supplementary file 1 — Additional file 1. [file 12909_2022_3360_MOESM1_ESM.docx]

**Pre-Questionnaire**

The following is an optional survey about bleeding and hemorrhage control. Your decision to complete the survey or not and your individual responses to the questions will have no impact on your grades and evaluations. We sincerely appreciate your contribution to this work.

1.What is your prior experience with bleeding/hemorrhage control?

○ No experience

○ Minimal basic training in hemorrhage control techniques

○ Formal training in hemorrhage control techniques

○ Have used hemorrhage control techniques on a live person

2.If you witnessed a mass casualty event tomorrow and saw someone with life-threatening femoral artery hemorrhage from an amputated leg, would you try to control the bleeding?

○ Yes

○ No

○ I don’t know

3. What is your reason(s) for NOT trying to control the bleeding?

○ I am afraid of blood

○ I would just not to get involved

○ I am not sure of what to do

○ Something else: ________

4.Have you ever compressed with fingers on a wound to control the bleeding?

○ Yes

○ No

5.Have you ever compressed with bandages to control bleeding?

○ Yes

○ No

6.Have you ever compressed with a tourniquet on someone?

○ Yes

○ No

7.How confident are you in following ability before classes?

Not at all(1) 2 3 4 very(5)

Compress with fingers ○ ○ ○ ○ ○

Compress with bandages ○ ○ ○ ○ ○

Compress with a tourniquet ○ ○ ○ ○ ○

8.How important is it for following groups of population to receive formal hemorrhage-control training?

Not at all(1) 2 3 4 very(5)

Medical graduates ○ ○ ○ ○ ○

Medical postgraduates ○ ○ ○ ○ ○

Doctors in the hospital ○ ○ ○ ○ ○

Hospital technicians, logisticians and administrators ○ ○ ○ ○ ○

General public ○ ○ ○ ○ ○

9.How important is it to have hemorrhage-control kits available in public areas, as AEDs are?

Not at all(1) 2 3 4 very(5)

○ ○ ○ ○ ○

10.Should formal hemorrhage-control training be incorporated into the medical school curriculum? If so, when?

○ It should not be taught during medical school

○ It should be optional

○ During the 1st year in medical school

○ During the anatomy course

○ During the surgery course

○ During the last year in internship

○ During the work in the hospital

11.What is the professional field you want to pursue in the future?

○ Internal Medicine

○ Surgery Medicine

○ Acute and Critical Care Medicine

○ Other Specialties

○ None-medical industry

○ I don’t know
