# Supplementary material for: Comparison of two teaching methods for stopping the bleed: a randomized controlled trial
Source: BMC Med Educ. 2022 Apr 14;22:281. doi: 10.1186/s12909-022-03360-4 (PMC9009024; doi:10.1186/s12909-022-03360-4)
Supplement: Supplementary file 2 — Additional file 2. [file 12909_2022_3360_MOESM2_ESM.docx]

**Post-Questionnaire**

The following is an optional survey about bleeding and hemorrhage control. Your decision to complete the survey or not and your individual responses to the questions will have no impact on your grades and evaluations. We sincerely appreciate your contribution to this work.

1.Now that you have received hemorrhage-control training, if you witnessed a mass casualty event tomorrow and saw someone with life-threatening femoral artery hemorrhage from an amputated leg, would you try to control the bleeding?

○ Yes

○ No

○ I don’t know

2.What is your reason(s) for NOT trying to control the bleeding?

○ I am afraid of blood

○ I would just not to get involved

○ I am not sure of what to do

○ Something else: ________

3.Now that you have received hemorrhage-control training, how confident are you in following ability?

Not at all(1) 2 3 4 very(5)

Compress with fingers ○ ○ ○ ○ ○

Compress with bandages ○ ○ ○ ○ ○

Compress with a tourniquet ○ ○ ○ ○ ○

4.How important is it for following groups of population to receive formal hemorrhage-control training?

Not at all(1) 2 3 4 very(5)

Medical graduates ○ ○ ○ ○ ○

Medical postgraduates ○ ○ ○ ○ ○

Doctors in the hospital ○ ○ ○ ○ ○

Hospital technicians, logisticians and administrators ○ ○ ○ ○ ○

General public ○ ○ ○ ○ ○

5.How important is it to have hemorrhage-control kits available in public areas, as AEDs are?

Not at all(1) 2 3 4 very(5)

○ ○ ○ ○ ○

6.Should formal hemorrhage-control training be incorporated into the medical school curriculum? If so, when?

○ It should not be taught during medical school

○ It should be optional

○ During the 1st year in medical school

○ During the anatomy course

○ During the surgery course

○ During the last year in internship

○ During the work in the hospital

7.What is the professional field you want to pursue in the future?

○ Internal Medicine

○ Surgery Medicine

○ Acute and Critical Care Medicine

○ Other specialties

○ None-medical industry

○ I don’t know

8.Do you have any interest in becoming a Stop the Bleed instructor?

○ Yes

○ No

9.After this training, do you feel yourself

Strongly disagree (1) 2 3 4 Strongly agree(5)

Teamwork skills were improved ○ ○ ○ ○ ○

Clinical thinking was improved ○ ○ ○ ○ ○

Problem analysis were improved ○ ○ ○ ○ ○

Scenario simulation / Operation enhanced learning

○ ○ ○ ○ ○

The distributed PowerPoint and study resources were helpful for learning

○ ○ ○ ○ ○

10.For this training, what do you think of the teacher?

Very dissatisfied (1) 2 3 4 Very satisfied(5)

Enthusiasm for lectures ○ ○ ○ ○ ○
Interaction with students ○ ○ ○ ○ ○

Overall teaching effect ○ ○ ○ ○ ○

11.What would you suggest for the course? Thanks for providing valuable advice.

______________________________________________
