# Supplementary material for: Comparison of two teaching methods for stopping the bleed: a randomized controlled trial
Source: BMC Med Educ. 2022 Apr 14;22:281. doi: 10.1186/s12909-022-03360-4 (PMC9009024; doi:10.1186/s12909-022-03360-4)
Supplement: Supplementary file 3 — Additional file 3. [file 12909_2022_3360_MOESM3_ESM.docx]

**Theoretical Test**

1.When using a tourniquet to stop bleeding, what are the details to pay attention to?

2.What are the meanings of ‘ABCDEF’ in trauma physical examination and ‘CRASH-PLAN’ in secondary assessment?

3.What is the meaning of ‘ABC’ in trauma hemostasis?
